# Differential effects of type 1 diabetes mellitus and subsequent osteoblastic β-catenin activation on trabecular and cortical bone in a mouse model

**DOI:** 10.1038/s12276-018-0186-y

**Published:** 2018-12-05

**Authors:** Sixu Chen, Daocheng Liu, Sihao He, Lei Yang, Quanwei Bao, Hao Qin, Huayu Liu, Yufeng Zhao, Zhaowen Zong

**Affiliations:** 1 State Key Laboratory of Trauma, Burn and Combined Injury, Department of War Wound Rescue Skills Training, Base of Army Health Service Training, Army Medical University, 400038 ChongQing, China; 2 Department of Orthopedics, The 118th Hospital of the Chinese People’s Liberation Army, 325000 Wenzhou, Zhejiang China; 3 Department of Emergency, Xinqiao Hospital, Army Medical University, 400037 ChongQing, China; 4 Department of Trauma Surgery, Daping Hospital, Army Medical University, 400042 ChongQing, China

## Abstract

Type 1 diabetes mellitus (T1DM) is a pathological condition associated with osteopenia. WNT/β-catenin signaling is implicated in this process. Trabecular and cortical bone respond differently to WNT/β-catenin signaling in healthy mice. We investigated whether this signaling has different effects on trabecular and cortical bone in T1DM. We first established a streptozotocin-induced T1DM mouse model and then constitutively activated β-catenin in osteoblasts in the setting of T1DM (T1-CA). The extent of bone loss was greater in trabecular bone than that in cortical bone in T1DM mice, and this difference was consistent with the reduction in the expression of β-catenin signaling in the two bone compartments. Further experiments demonstrated that in T1DM mice, trabecular bone showed lower levels of insulin-like growth factor-1 receptor (IGF-1R) than the levels in cortical bone, leading to lower WNT/β-catenin signaling activity through the inhibition of the IGF-1R/Akt/glycogen synthase kinase 3β (GSK3β) pathway. After β-catenin was activated in T1-CA mice, the bone mass and bone strength increased to substantially greater extents in trabecular bone than those in cortical bone. In addition, the cortical bone of the T1-CA mice displayed an unexpected increase in bone porosity, with increased bone resorption. The downregulated expression of WNT16 might be responsible for these cortical bone changes. In conclusion, we found that although the activation of WNT/β-catenin signaling increased the trabecular bone mass and bone strength in T1DM mice, it also increased the cortical bone porosity, impairing the bone strength. These findings should be considered in the future treatment of T1DM-related osteopenia.

## Diabetes: Bone types respond differently to potential intervention

Activation of a key signaling pathway implicated in bone mass maintenance helps boost the strength of spongy internal bone in a mouse model of type 1 diabetes mellitus (T1DM) but also increases the porosity of the outer surface, compromising bone integrity. Zhaowen Zong and colleagues from the Army Medical University in ChongQing, China experimentally induced T1DM in mice and saw greater loss of inner ‘trabecular’ bone than outer ‘cortical’ bone. Looking to improve bone strength, the researchers genetically activated a key signaling pathway by boosting β-catenin levels in bone-forming osteoblast cells. This intervention had different effects on the two bone types, with increased mass and strength in trabecular bone but increased porosity and fragility in cortical bone. The findings offer a cautionary note that therapeutically elevating WNT/β-catenin signaling may not enhance overall bone function in patients with T1DM.

## Introduction

The autoimmune destruction of insulin-producing β-cells in the pancreas causes type 1 diabetes mellitus (T1DM). This leads to complete insulin deficiency and a concomitant disruption of glucose homeostasis. T1DM patients have twice the risk of any fracture and a 4–5-fold greater risk of hip fracture than individuals without diabetes^1^. Furthermore, the outcomes of their fractures are worse, and these individuals are more likely to experience delayed healing and postsurgical complications, including wound infection^2–4^.

The canonical WNT signaling pathway regulates gene transcription by stabilizing β-catenin, and it has been implicated in a wide range of physiological and pathophysiological processes^5,6^. Accumulating evidence suggests that the interactions between glucose metabolism and WNT/β-catenin signaling are reciprocal. Several studies have suggested the potential involvement of WNT signaling in regulating glucose metabolism^7–9^. Another study demonstrated mice that lack low-density lipoprotein receptor-related protein 5 (LRP5), a coreceptor for WNT signaling, showed markedly impaired glucose tolerance as a result of the impairment of glucose-induced insulin secretion^10^. Moreover, diabetes mellitus and hyperglycemia affect the WNT signaling pathway and, in turn, impair bone metabolism. It is well established that bone formation is impaired in both patients with T1DM and streptozotocin (STZ)-induced diabetic rodents, which could be the primary cause of T1DM-induced bone loss. However, various studies have shown that bone resorption is unchanged, increased, or reduced in different diabetic states^11–13^.

WNT/β-catenin signaling is essential for osteoblast proliferation and differentiation, and it has long been proven to participate in bone modeling and remodeling^14^. Several recent studies have demonstrated that a reduction in WNT/β-catenin signaling impairs osteoblast activity in T1DM^11,15,16^. Therefore, the manipulation of the WNT/β-catenin signaling pathway may be an ideal treatment option for T1DM-associated bone loss^11,17,18^.

Trabecular bone and cortical bone differ in their structures, mechanical properties, and metabolic activities^19–21^. If they are considered two different entities, the clinical outcomes of treating the respective pathologies of each compartment should be improved^22–26^. The expression levels of WNT/β-catenin-signaling-related molecules have been reported to differ in trabecular and cortical bone in healthy mice. Trabecular bone is more readily affected by changes in the WNT/β-catenin signaling pathway than cortical bone^27^. Other studies have also reported that the effects of WNT ligands differ between trabecular bone and cortical bone. For example, WNT5a and WNT10b promoted osteogenesis with an increased bone mass in the trabecular bone, with no effect on the cortical bone^28,29^, whereas WNT16 reduced bone resorption on the endocortical surface but not in the trabecular bone^30^. Taken together, these findings indicate that trabecular and cortical bone react differently to WNT/β-catenin signaling pathway manipulations. Consequently, we hypothesized that the deleterious effects of T1DM on cortical bone and trabecular bone differ and that this difference is related to the different expression levels of WNT/β-catenin signaling in the two bone types. The manipulation of WNT/β-catenin signaling may also differentially affect trabecular and cortical bone in T1DM.

Therefore, in this study, we investigated whether trabecular and cortical bone respond differently to the diabetic state in a T1DM mouse model and examined the effects of the osteoblastic activation of β-catenin on trabecular and cortical bone within the context of T1DM.

## Materials and methods

### Animal models

For the stabilization of β-catenin in committed osteoblasts, Col1-3.2kb-Cre^ERTM^; Catnblox(ex3) mice were initially generated and then intraperitoneally injected with tamoxifen (TM; Sigma-Aldrich, St Louis, MO, USA) to activate the function of the promoter, as previously reported by our team^31,32^. However, we used a lower dose of TM than that used in previous studies, at 10 mg/kg/day × 4 consecutive days, which has been proven to yield a similar Cre induction efficacy with minimum bone-active side effects in young male mice^33^. To determine the site-specificity of Col1-3.2kb-Cre^ERTM^, ROSA26 mice were crossed with Col1-3.2kb-Cre^ERTM^ transgenic mice, and β-galactosidase (β-gal) staining was performed as reported^34^ to examine the expression pattern of Col1-3.2kb-Cre^ERTM^ after treatment with tamoxifen injection.

Col1-3.2kb-Cre^ERTM^; Catnblox(ex3) mice and littermate controls were used in the experiments. All male mice aged 8 weeks were allotted to three groups of 10 mice each as follows: Control group with phosphate-buffered saline (PBS) injection in littermate control mice; T1DM group with STZ injection in littermate control mice; and T1-CA group with STZ injection plus osteoblastic β-catenin activation in Col1-3.2kb-Cre^ERTM^; Catnblox(ex3) mice. STZ (50 mg/kg; Sigma) or PBS was administered by intraperitoneal injection once per day for the first 6 days. Seven days after the last injection, the nonfasting blood glucose levels were measured in blood collected from the mouse tail, and mice with blood glucose levels greater than 300 mg/dl were considered diabetic. The T1-CA mice were then intraperitoneally injected with TM (10 mg/kg) for four consecutive days to activate β-catenin in osteoblasts. Six weeks after the first injection of STZ or PBS, all mice were euthanized and dissected to obtain the bilateral femurs and tibiae. Bone samples were fixed in formalin or frozen in liquid nitrogen and stored at −80 °C. At this time, the body weight and femur length were measured. The cage bedding and water supply of the diabetic mice were frequently replaced. All animal procedures were performed in accordance with protocols approved by the Third Military Medical University Institutional Animal Care and Use Committee.

### X-ray imaging and microcomputed tomography (micro-CT) examination

Radiographic images of fixed femurs in 70% ethanol were obtained using a MX-20 Cabinet X-ray system (Faxitron X-ray Corporation). The femurs were subsequently subjected to micro-CT analysis using a viva CT 40 system (Scanco Medical AG) following the procedural recommendations of the American Society for Bone and Mineral Research^35^. The scanning medium was ethanol, the X-ray tube potential was 45 kVp, and the voxel size was 10 mm^−3^. Images were reconstructed and analyzed using a global threshold of 1400 Hounsfield units. Quantitative morphometric data were based on the region of interest as follows: the trabecular bone region was defined at 0.17 mm under the growth plate of the distal femur extending 2 mm toward the diaphysis and excluding the outer cortical shell. Cortical bone measurements were obtained in a region that started 50% of the bone length below the femoral head and extended 1.2 mm. The bone volume per total volume (BV/TV), trabecular thickness (Tb.Th.), number (Tb.N.), and separation (Tb.Sp.) were calculated for trabecular bone, whereas the cortical bone area per tissue area (BA/TA) and cortical thickness (CT.Th.) were quantified from cortical bone.

### Bone histological analysis and histomorphometry

Mice were euthanized at the indicated ages; the tibiae and femora were fixed in 4% neutral-buffered formalin at 4 °C overnight, decalcified in 0.5 M EDTA for 10 days, embedded in paraffin, and sectioned at 4 μm thickness. To assess the tissue morphology, sections were stained with H&E as previously reported^34^. Tartrate-resistant acid phosphatase staining was performed to evaluate the osteoclast activity and bone resorption^34^.

For dynamic histomorphometric analysis, the mice were injected with calcein (30 mg/kg; Sigma-Aldrich) and alizarin red (50 mg/kg; Sangon Biotech) 7 and 2 days prior to euthanasia. The femurs were subsequently dissected free of tissue, fixed in ethanol, and embedded in methyl methacrylate. Transverse sections of the midshaft femur and longitudinal sections of the distal femur were cut to 20 and 5 μm, respectively, using a large scale, heavy duty sectioning system (Leica SP2600 Ultramiller). Trabecular and endocortical bone formation parameters were measured from the calcein and alizarin labels. For static histomorphometric analysis, TRAP staining sections were used to measure osteoblast and osteoclast-related parameters. Analysis was performed using the OsteoMeasure morphometric system (OsteoMetrics Inc.). The terminology and units employed are recommended by the Histomorphometry Nomenclature Committee of the American Society for Bone and Mineral Research^36^. To evaluate bone mineralization, undecalcified 5 μm femur sections were analyzed with Von Kossa staining as previously described^34^. Further quantification of the mineralization level was assessed by measuring the optical density of the mineralized bone area, using ImageJ 1.51 (National Institutes of Health, USA). To assess the cortical porosity, the same OsteoMeasure morphometric system was employed to measure the cortical bone area and porotic area (canal area) in the femoral cortical region from the same sections used to derive the bone formation rates. These data were employed to calculate the cortical porosity (total porotic area/cortical bone area × 100, %) in accordance with the method of Power et al.^37^.

### Immunohistochemical staining

Immunohistochemistry was performed as previously described^34^. The primary antibodies used in the study included a rabbit monoclonal anti-β-catenin antibody (1:400; #8814; Cell Signaling), a rabbit polyclonal anti-Dickkopf-1 antibody (1:100; sc-25516; Santa Cruz), a mouse monoclonal anti-WNT16 antibody (1:100; sc-271897; Santa Cruz), and a rabbit polyclonal anti-WNT5a antibody (1:100; ab72583; Abcam). The biotinylated goat anti-mouse and goat anti-rabbit IgG were acquired from Boster (Wuhan, China). To obtain the percentage of cells that expressed a given marker protein, photomicrographic images of each section were captured with an Olympus microscope and a digital camera under ×200magnification. The number of specific antigen-positive cells was counted in five random fields. The mean and standard deviation of the percentage of positive cells were calculated for each group and were used for the statistical analysis.

### Mechanical testing

The mechanical strength of the femoral diaphysis was assessed using the three-point bending test with a BOSE ElectroForce ELF 3200 computer-controlled testing machine as previously described^34^. Several parameters, including the stiffness, work to failure, and ultimate force, were analyzed. The distal metaphysis of the femur was subsequently severed at a length of 5 mm from the joint surface. The mechanical strength of the trabecular bone was then measured using a materials-testing machine (ElectroPuls). A compressive load was applied using a rectangular parallelepiped mold on the femoral distal metaphysis from the lateral to medial aspect. The loading point was directed to the center of the femoral lateral condyle. Load–displacement curves were recorded at a compression speed of 5 mm/min and a compression depth of 2.5 mm. The parameters analyzed included the stiffness and ultimate force.

### In vitro studies using MC3T3-E1 cells and primary mouse osteoblast cells

Mouse osteoblastic MC3T3-E1 subclone four cells (CRL-2593; American Type Culture Collection) were cultured in α-MEM, 10% fetal bovine serum (FBS), and 1% penicillin–streptomycin. To evaluate the effect of a high-glucose environment on the expression of the indicated pathway genes, cells were incubated with normal (5.5 mM) or high (25 mM) glucose for a 24 h-period.

Primary mouse osteoblasts were isolated from the long bones of Catnblox(ex3) mice by sequential digestions as previously described^34^. Briefly, the bone pieces were incubated with 4 mL of collagenase solution at 37 °C in a shaking water bath for 2 h to remove all remaining soft tissue and adhering cells; the samples were then transferred to medium that contained 10% FBS to inhibit further collagenase activity, rinsed three times with medium, and transferred to 25 cm^2^ flasks at a density of approximately 20–30 fragments per flask. The culture medium was replaced three times per week. The primary osteoblasts from trabecular and cortical bones were cultured separately. Cells were first incubated with high (25 mM) glucose for 12 h. β-Catenin was subsequently activated by the addition of Cre adenovirus for an additional 12 h. The protein concentrations of WNT16 and WNT5a were measured in the culture medium using Mouse Protein WNT16 and WNT5a ELISA kits (CUSABIO), respectively.

### RNA extraction and quantitative PCR

Femurs were cleaned of muscle and connective tissue; the growth plates along with the articular cartilage were removed, and the metaphyses and diaphyses were separated by cutting between the boundary, which was judged by the transition from red to white in color. The bone marrow was subsequently removed via saline flush. Specimens of metaphyses and diaphyses were cut into cubic structures with the same apparent volumes (approximately equal to 2 × 2 × 2 mm^3^). The metaphyses and diaphyses were then snap frozen in liquid nitrogen and separately stored at −80 °C. Frozen samples were crushed in liquid nitrogen, and total RNA was extracted from the bone samples or cultured cells using TRIzol reagent (Ambion/Life Technologies) according to the manufacturer’s instructions. The mRNA expression levels were tested using the SYBR green detection method (Bio-Rad Laboratories). β-Actin served as a control, and the expression levels of a given gene were expressed as the proportion relative to the mean β-actin value. The primers are listed in Table S1.

### Western blot analysis

For protein analysis, cells were washed in PBS and cellular proteins were extracted in RIPA buffer (Beyotime Institute of Biotechnology) for 30 min at 4 °C. Lysates were cleared by centrifugation, and the resulting supernatant was transferred to a new tube. The protein concentration of the supernatant was quantified with a BCA Protein Assay Reagent Kit (Beyotime Institute of Biotechnology). The whole-protein extracts were then discontinuously separated on sodium dodecyl sulfate-polyacrylamide gels and transferred to polyvinylidene fluoride membranes. After blocking, the membranes were incubated overnight at 4 °C with the rabbit monoclonal Anti-GSK3 beta (phospho S9) antibody (1:10000; ab75814; Abcam), rabbit monoclonal anti-β-catenin (1:1000; #8814), anti-IGF-1R (1:1000; #8521), or anti-Phospho-Akt (1:2000; #4060) antibodies (Cell Signaling Technology). The membranes were subsequently incubated with the corresponding horseradish peroxidase-conjugated secondary antibodies for 1 h (1:5000; GE Healthcare). Protein expression was visualized using luminol-enhanced chemiluminescence and was densitometrically assessed. β-Actin served as a control, and the expression levels of a given protein are expressed as the proportion relative to the mean β-actin value. The protein bands were quantified using Quantity One software (Bio-Rad).

### Statistical analysis

All data were normally distributed, exhibited equivalent variances, and are expressed as the mean ± standard deviation (SD). A probability (*p*) value of <0.05 or 0.01 was considered statistically significant. Statistical analysis was performed using Microsoft Excel 2010 (Microsoft Corporation) and SigmaStat (SPSS). Group mean values were compared by Student’s two-tailed *T*-tests.

## Results

### Bone loss was more striking in trabecular bone than that in cortical bone in STZ-induced T1DM mice

**Fig. 1:**
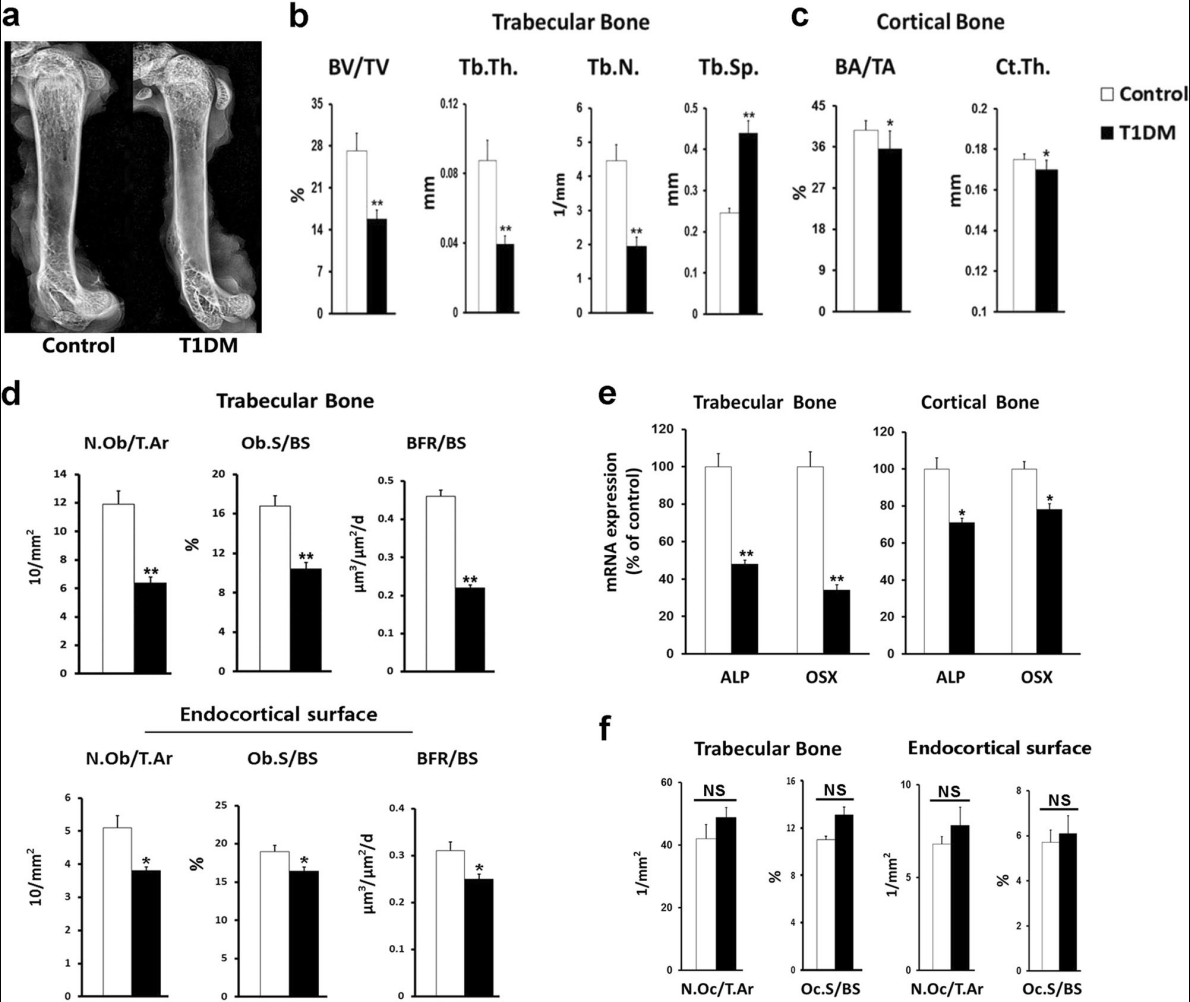
Streptozotocin (STZ)-induced T1DM mice showed a greater extent of osteopenia in cancellous bone than in cortical bone. **a** Representative X-ray images of mouse femur. **b**, **c** Trabecular and cortical parameters, including bone volume per total volume (BV/TV), trabecular thickness (Tb.Th.), trabecular number (Tb.N.), trabecular separation (Tb.Sp.), cortical bone area per tissue area (BA/TA), and cortical thickness (CT.Th.), as measured by micro-CT. *n* = 9/control, 7/T1DM. **d** Static and dynamic histomorphometric analyses were performed in the trabecular bone and endocortical surface of the femur. N.Ob/T.Ar osteoblast number per tissue area, Ob.S/BS osteoblast surface per bone surface, BFR/BS bone formation rate per bone surface. *n* = 9. **e** mRNA expression levels of osteoblast markers in trabecular and cortical bone. ALP alkaline phosphatase, OSX osterix. *n* = 8. **f** Histomorphometric quantification of TRAP-stained osteoclasts in the trabecular bone and endocortical surface of the femur. *n* = 9. N.Oc/T.Ar osteoclast number normalized to tissue area, Oc.S/BS osteoclast surface normalized to bone surface. Data are expressed as the mean ± SD. *P < 0.05, **P < 0.01 versus Control group by an unpaired *t*-test. NS not significant, *P* > 0.05

The T1DM mice had lower bodyweights than those of the control mice at the end of the experiment; however, they did not significantly differ in the femur length or bone shape (Fig. S1 and Fig. 1a). The bone density was lower in the femurs of the T1DM mice than in those of the control mice, mainly at the distal and proximal femoral metaphyses rather than at the femoral diaphyses (Fig. 1a). Microcomputed tomography also showed different extents of bone loss in the trabecular and cortical bone (Fig. 1b, c). The trabecular parameters (bone volume per total volume [BV/TV], trabecular thickness [Tb.Th.], and trabecular number [Tb.N]) were significantly lower in the T1DM mice than those in the control mice (reductions of 41.6%, 55%, and 56.3%, respectively), whereas the corresponding cortical parameters (bone area per tissue area [BA/TA] and cortical thickness [CT.Th.]) decreased to a substantially less extent (reductions of 10.4% and 2.8%, respectively) in the T1DM mice compared with the control mice. A bone histomorphometric analysis demonstrated reduced osteoblast activity and bone formation in both the trabecular and cortical bones of the T1DM mice. The reductions in the osteoblast number per tissue area (N.Ob/T.Ar), osteoblast surface per bone surface (Ob.S/BS), and bone formation rate (BFR) were also greater in the trabecular bone (46.2%, 38.1%, and 52.2%, respectively) than those on the endocortical surface (25.5%, 13.7%, and 19.4%, respectively) (Fig. 1d). The expressions of genes that encode the osteoblast markers alkaline phosphatase and osterix (OSX) were also lower in the T1DM mice than those in the control mice. Similarly, the extent of these reductions was more striking in the trabecular bone than that in the cortical bone (Fig. 1e). With respect to bone resorption, only a slight increase in the number of osteoclasts was identified in the T1DM mice compared with the control mice, and the incremental changes were not different between the trabecular bone and the endocortical surface (Fig. 1f).

### More pronounced downregulation of WNT/β-catenin signaling in trabecular bone than that in cortical bone was associated with the different effects of T1DM on the two bone compartments

**Fig. 2:**
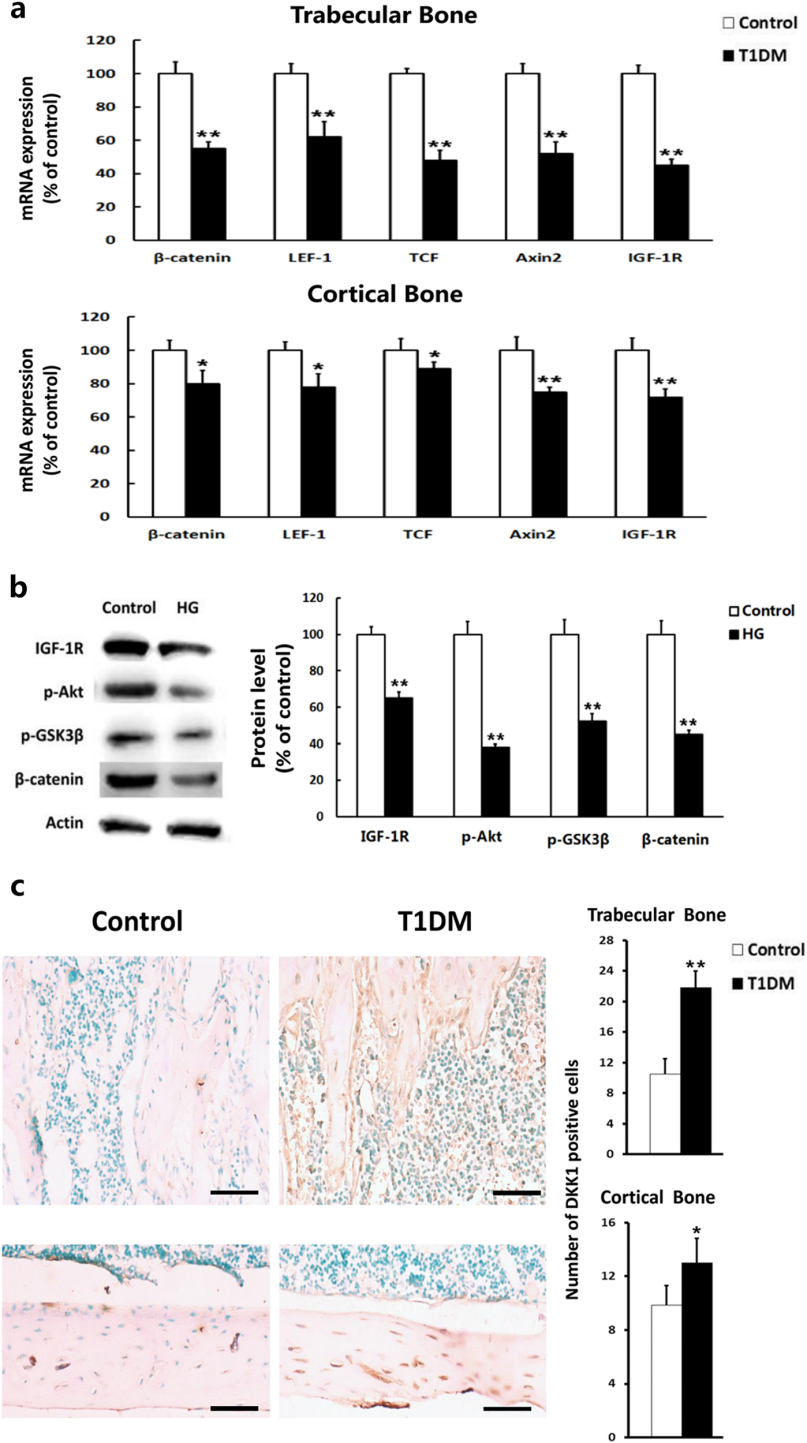
T1DM mice exhibited greater downregulated expression level of WNT/β-catenin signaling in trabecular bone than in cortical bone through inhibition of IGF-1R/Akt/GSK3β pathway. **a** mRNA expression levels of IGF-1R, β-catenin, and canonical WNT target genes in trabecular and cortical bone. LEF1 lymphoid enhancer factor-1, TCF T cell factor, Axin2 axis inhibition protein 2, IGF-1R insulin-like growth factor-1 receptor. *n* = 8. **b** Western blots of IGF-1R, β-catenin, phosphorylated Akt, and GSK3β in MC3T3-E1 cells exposed to normal glucose (control) or high glucose (HG). The protein levels were quantified by densitometry and represented graphically. *n* = 8. **c** Dickkopf-1 protein expression was detected by immunohistochemistry in longitudinal sections of femoral trabecular and cortical bone. The quantitative results are shown on the right. *n* = 9. Scale bars, 40 μm. DKK-1 Dickkopf-1. Data are expressed as the mean ± SD. *P < 0.05, **P < 0.01 versus Control group by an unpaired *t*-test

The inhibition of canonical WNT/β-catenin signaling is considered a potential mechanism of the bone loss observed in T1DM^11,38^. Therefore, the expression levels of β-catenin and other canonical WNT-targeted genes, including genes that encode lymphoid enhancer factor-1 (LEF1), T cell factor (TCF), and axis inhibition protein 2 (Axin2), were examined in the trabecular and cortical bone. The expressions of these genes were downregulated in both the trabecular and cortical bone in the T1DM mice, and the reductions in the expression of β-catenin, LEF1, TCF, and Axin2 were more striking in the trabecular bone (45%, 38%, 52%, and 48%, respectively) than those in the cortical bone (20%, 22%, 11%, and 25%, respectively) (Fig. 2a).

The mechanism that underlies the different effects of T1DM on trabecular and cortical bone was subsequently investigated. The high glucose or low insulin levels in T1DM were found to reduce the level of insulin-like growth factor-1 receptor (IGF-1R) in osteoblasts, which might, in turn, downregulate the expression of β-catenin through the IGF-1R/AKT/GSK3β pathway^11,18^. Here, we also found that the reduction in the expression of IGF-1R was greater in the trabecular bone than that in the cortical bone (45% vs. 28%, respectively) (Fig. 2a). When cultured in the high-glucose environment, the level of IGF-1R protein decreased in osteoblastic MC3T3-E1 cells, together with reductions in phosphorylated AKT, phosphorylated GSK3β, and β-catenin protein (Fig. 2b).

Moreover, we measured the expression level of Dickkopf-1, a soluble glycoprotein that inhibits the WNT signaling pathway by binding to LRP5 and LRP6 (ref. ^39^). The protein expression of Dickkopf-1 was higher in the T1DM mice than that in the control mice, and the extent of the increase was greater in the trabecular bone than that in the cortical bone (Fig. 2c).

### Osteoblastic activation of β-catenin in T1DM mice abrogated bone loss and improved bone strength in the trabecular bone

Because the downregulation of WNT/β-catenin signaling may be a crucial factor in T1DM-induced bone loss, we subsequently investigated whether the activation of β-catenin increases the bone mass in T1DM mice. β-Catenin was conditionally activated in osteoblasts of Col1-3.2kb-Cre^ERTM^; Catnblox(ex3) mice by injecting tamoxifen. To confirm the Cre activity in osteoblasts, Col1-3.2kb-Cre^ERTM^ mice were mated with ROSA26 reporter mice, and when the Col1-3.2kb-Cre^ERTM^; ROSA26 mice were treated with tamoxifen, they displayed a strong positive signal in most cortical and trabecular surface osteoblasts as assessed by β-galactosidase staining (Fig. S2). There were no positive signals in the mice treated with vehicle (Fig. S2). Furthermore, immunohistochemical staining showed that the osteoblasts positive for β-catenin were significantly greater in the tamoxifen-treated Col1-3.2kb-Cre^ERTM^; Catnblox(ex3) mice than those in the mice treated with vehicle (Fig. S3). These findings indicated that the inducible Cre model was capable of recombining and activating β-catenin in osteoblasts in vivo.

**Fig. 3:**
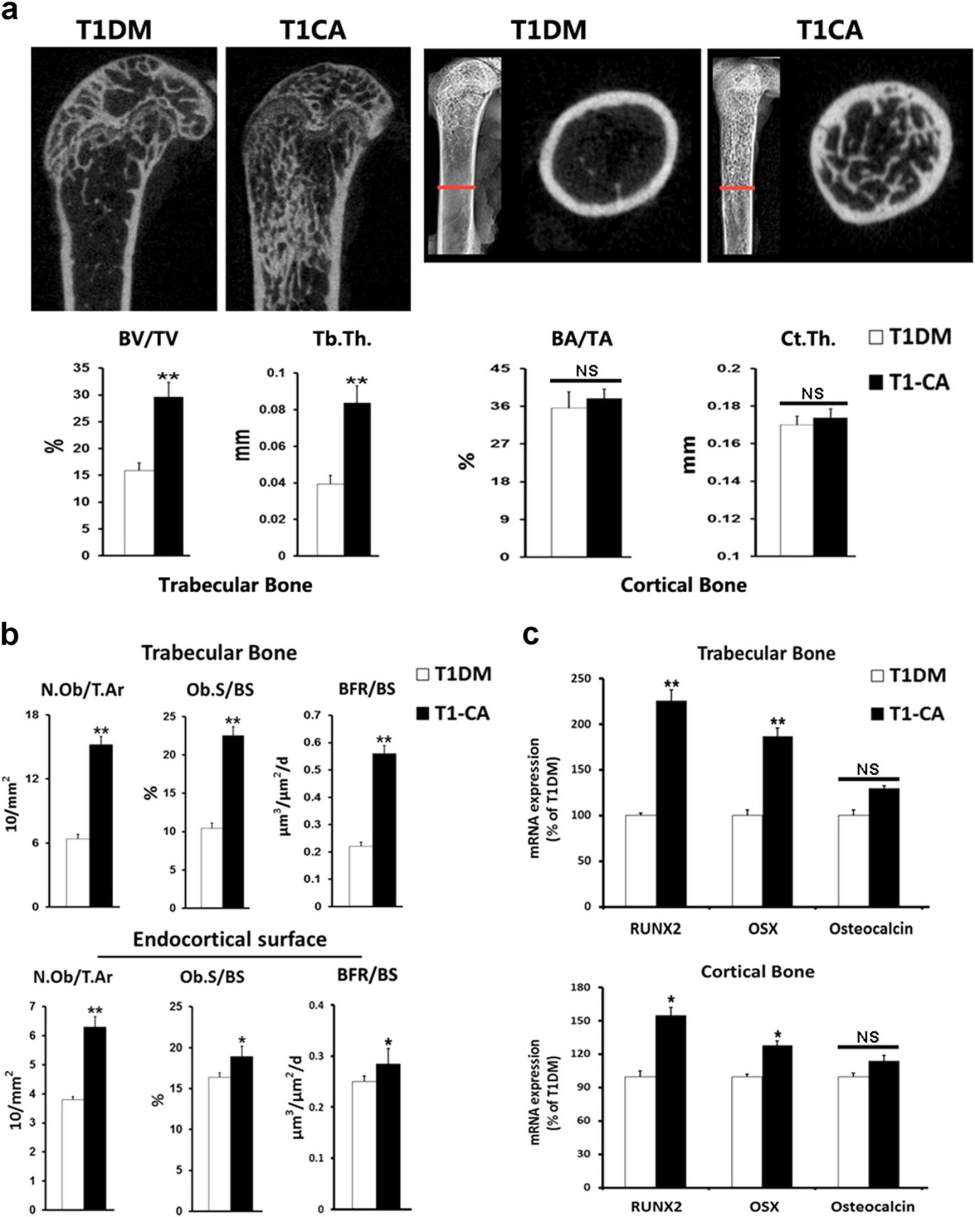
Osteoblastic activation of β-catenin in T1DM mice led to greater extent of bone mass increment and increase of osteogenic activities in trabecular bone compared with cortical bone. **a** Representative Micro-CT longitudinal reconstructed images of distal femur and cross-sectional images of the femoral diaphysis. BV/TV, Tb.Th (Trabecular bone), BA/TA, and Ct.Th (Cortical bone) are shown. *n* = 9/control, 7/T1DM. **b** Static and dynamic histomorphometric analyses in the trabecular bone and endocortical surface of the femur. *n* = 9. **c** mRNA expression levels of osteoblast markers in trabecular and cortical bone. *n* = 8. Data are expressed as the mean ± SD. *P < 0.05, **P < 0.01 versus T1DM group by an unpaired *t*-test. NS not significant, *P* > 0.05

**Fig. 4:**
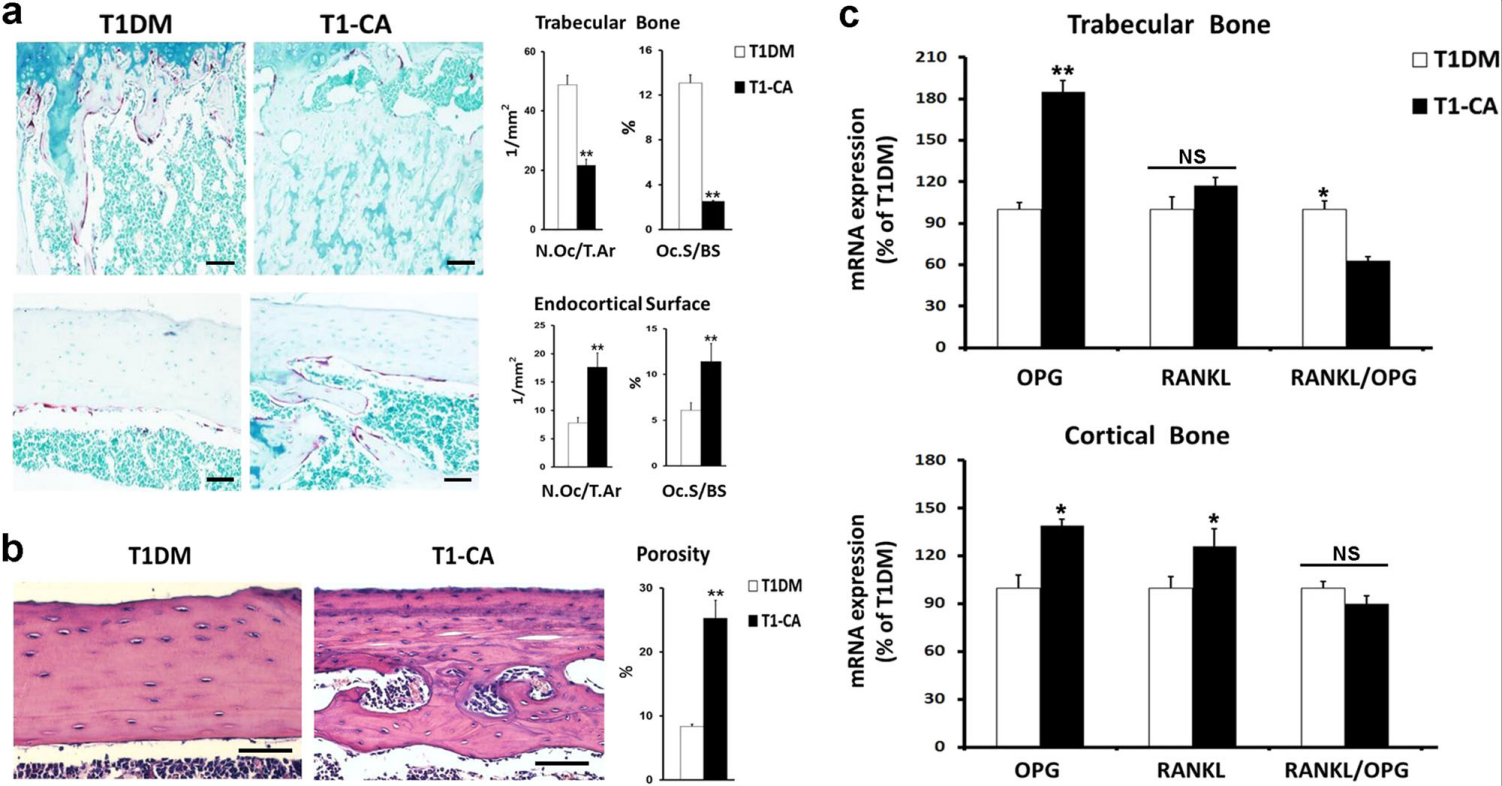
Osteoblastic activation of β-catenin in T1DM mice increased osteoclasts activities and bone porosity in cortical bone but not in trabecular bone. **a** Representative images of TRAP staining and histomorphometric quantification of TRAP-stained osteoclasts in femoral trabecular and cortical bone. *n* = 9. Scale bars, 50 μm. **b** Representative images of H&E-stained longitudinal sections of the femoral diaphysis. The cortical porosity was quantified by histomorphometry and represented graphically. *n* = 9. Scale bars, 40 μm. **c** mRNA expression levels of osteoclast regulating factors in trabecular and cortical bone. *n* = 8. RANKL receptor activator of nuclear kappa B ligand, OPG osteoprotegerin. Data are expressed as the mean ± SD. *P < 0.05, **P < 0.01 versus T1DM group by an unpaired *t*-test. N, not significant, *P* > 0.05

The trabecular bone mass increased after β-catenin was activated in the osteoblasts in the diabetic state (T1-CA mice), and the increments in the BV/TV and Tb.Th were 86.7% and 112.5%, respectively (Fig. 3a and Fig. S4). A histomorphometric analysis indicated an increased osteoblast number and surface in the trabecular bone of the T1-CA mice (Fig. 3b). Accordingly, the bone formation rate increased by 154.5% after β-catenin activation (Fig. 3b). Consistent with the changes in bone formation, the expressions of the genes that encode RUNX2 and OSX, two markers for the early differentiation of osteoblasts, increased in the trabecular bone, with no change identified in the expression of osteocalcin (Fig. 3c), a marker of fully differentiated osteoblasts. In addition to greater bone formation, fewer osteoclasts were identified in the trabecular bone of the T1-CA mice, when examined with osteoclast-specific TRAP staining and histomorphometric quantification (Fig. 4a). This effect was also associated with a higher expression of osteoprotegerin (OPG) and a lower ratio of receptor activator of nuclear kappa B ligand (RANKL) to OPG in the trabecular bone (Fig. 4c).

**Fig. 5:**
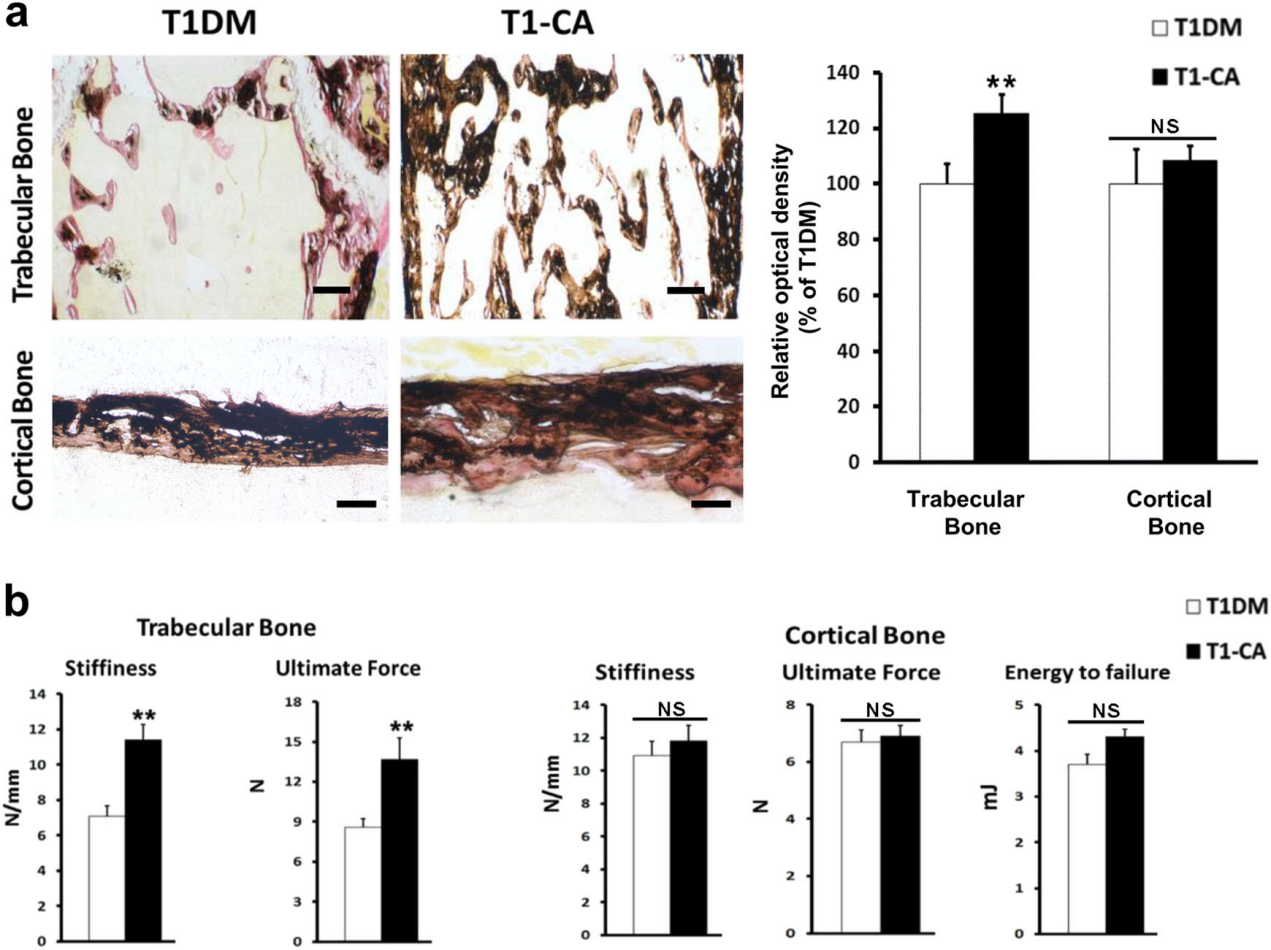
Osteoblastic activation of β-catenin in T1DM mice improved bone strength to a greater extent in trabecular bone than in cortical bone. **a** Representative images of Von Kossa staining of femoral trabecular and cortical bone, with quantitative analysis on the right. *n* = 9. Scale bars, 50 μm. **b** Mechanical strength of the femoral distal metaphysis and femoral diaphysis, as measured by a compression test and three-point bending test, respectively. *n* = 9. Data are expressed as the mean ± SD. **P < 0.01 versus T1DM group by an unpaired *t*-test. NS not significant, *P* > 0.05

The bone mineralization and bone strength were also assessed in the trabecular bone. Von Kossa staining showed more mineralized bone in the distal femurs of the T1-CA mice than in those of the T1DM mice (Fig. 5a). Moreover, the T1-CA mice displayed increased stiffness and ultimate force in the femoral distal metaphysis than that in the T1DM mice when a compression test was applied (Fig. 5b).

### Effects of osteoblastic activation of β-catenin in T1DM mice on cortical bone differed from those on trabecular bone

Osteoblastic activation of β-catenin in T1DM mice had different effects on cortical and trabecular bone. After β-catenin was activated in osteoblasts, the cortical bone mass increased in the T1-CA mice compared with that in the T1DM mice but to a lesser extent than that in the trabecular bone (Fig. 3a). Similar trends were identified in the osteoblast activity, BFR, and expressions of RUNX2 and OSX (Fig. 3b, c).

The most striking difference was that more TRAP-positive cells were identified on the endocortical surface of the cortical bone. TRAP staining and quantification indicated that the numbers and surfaces of osteoclasts on the femoral endocortical surfaces of the T1-CA mice increased by 125.6% and 86.9%, respectively (Fig. 4a). This increase was associated with a significant (201.2%) increase in the cortical porosity at the femoral diaphysis in the T1-CA mice compared with that in the T1DM mice (Fig. 4b).

Von Kossa staining showed that an improvement in bone mineralization was not present at the femoral diaphysis in the T1-CA mice (Fig. 5a). According to a three-point bending analysis of the femoral diaphysis, the cortical bone strength was slightly higher in the T1-CA mice than that in the T1DM mice; however, they were not significantly different (Fig. 5b). The extents of the increases in the bone stiffness and ultimate force were substantially smaller in the cortical bone than those in the trabecular bone (Fig. 5b).

### Potential mechanisms underlying increased cortical porosity

The expressions of RANKL and OPG, which are implicated in osteoclastogenesis, were examined. The T1-CA mice displayed a higher expression of OPG and a lower ratio of RANKL to OPG in the cortical bone than that in the T1DM mice, and these parameters were similar to those in the trabecular bone (Fig. 4c).

**Fig. 6:**
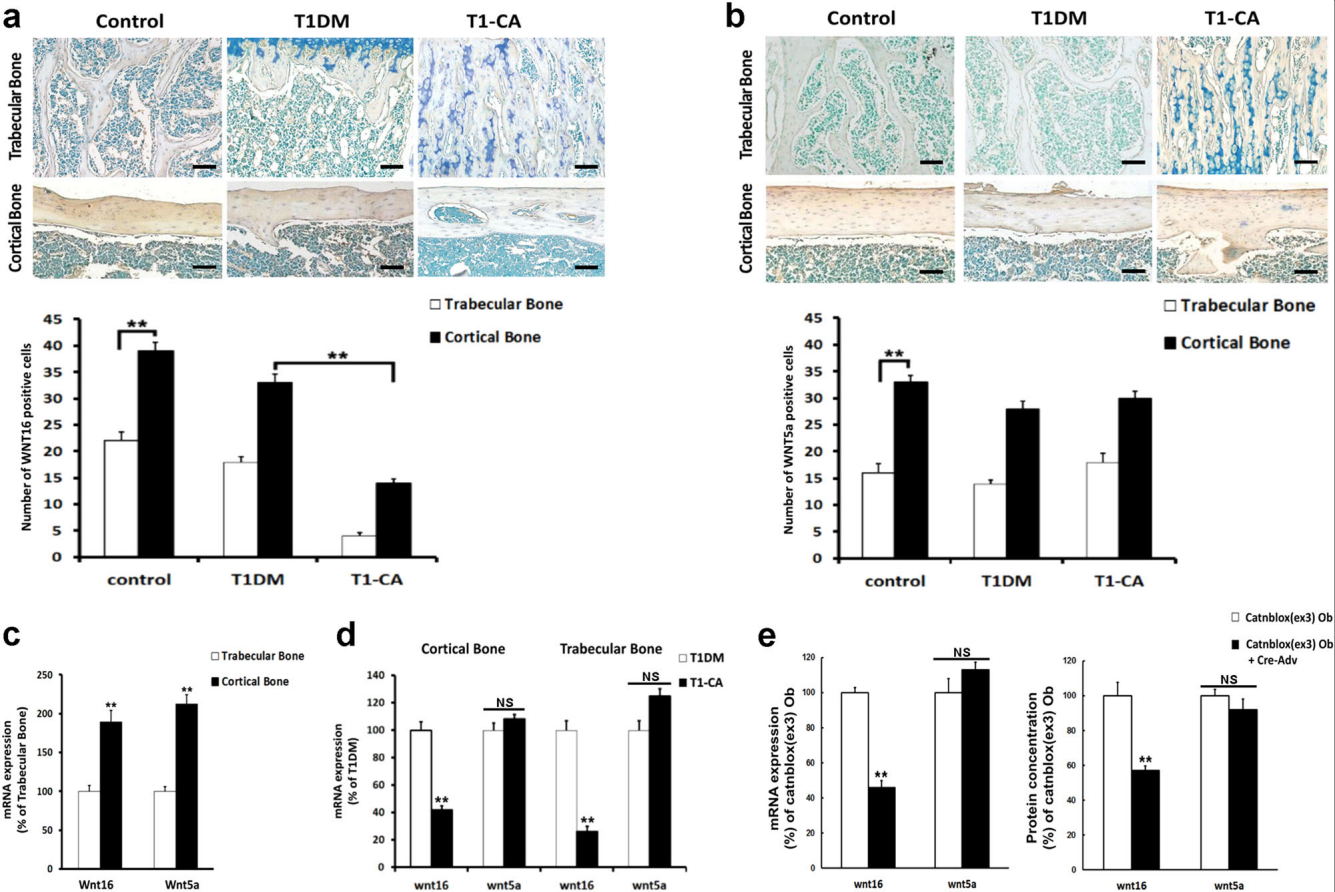
Downregulated expression of WNT16 was responsible for the unexpected increased bone resorption in cortical bone of T1-CA mice. **a** WNT16 and **b** WNT5a protein expressions were detected by immunohistochemistry in longitudinal sections of femoral trabecular and cortical bone. The quantitative results are shown. *n* = 9. Scale bars, 50 μm. **c** mRNA expression levels of WNT16 and WNT5a in trabecular and cortical bone of control mice. *n* = 8. **d** mRNA expression levels of WNT16 and WNT5a in trabecular bone and cortical bone in T1DM and T1-CA mice. *n* = 8. **e** mRNA expression levels and protein concentrations of WNT16 and WNT5a in primary osteoblasts from Catnblox(ex3) mice treated with high glucose plus Cre adenovirus or vehicle. *n* = 8. Ob osteoblast. Data are expressed as the mean ± SD. **P < 0.01 versus trabecular bone (**a**–**c**), T1DM group (**d**) or Catnblox(ex3) osteoblasts (**e**) by an unpaired *t*-test. NS not significant, *P* > 0.05

WNT16 and WNT5a have been reported to inhibit or enhance osteoclastogenesis, respectively, and both are highly expressed in adult cortical bone^29,30^. Our immunohistochemical results indicated that the numbers of osteoblasts positive for WNT16 and WNT5a were substantially greater in the cortical bone than those in the trabecular bone in both the control mice and T1DM mice (Fig. 6a, b). The WNT16 expression was slightly reduced in the cortical bone of the T1DM mice compared with that in the control mice (Fig. 6a); however, no difference was identified in the expression of WNT5a (Fig. 6b). Moreover, the expression of WNT16 significantly decreased (Fig. 6a) in the cortical bone of the T1-CA mice compared with that in the T1DM mice, whereas WNT5a remained unchanged (Fig. 6b). Furthermore, almost no WNT16-positive cells were identified in the trabecular bone of the T1-CA mice (Fig. 6a). These changes were confirmed with a mRNA expression analysis (Fig. 6c, d). Primary osteoblasts were then isolated from the long bones of Catnblox(ex3) mice and exposed to a high-glucose environment. β-Catenin was activated by Cre adenovirus infection, and both the mRNA expression and protein concentration of WNT16 decreased (Fig. 6e). No changes were identified in the WNT5a mRNA level and protein concentration (Fig. 6e).

## Discussion

Accumulating evidence indicates that trabecular and cortical bone have different metabolic behaviors during many physiological and pathological processes, including osteoporosis, glycation, pharmacokinetic responses, and reactions to diet, drugs, and implant insertion^22,25,26^. Research efforts in the field of osteoporosis have focused on the pathogenesis and prevention of accelerated trabecular bone loss over long periods. However, cortical bone loss was recently implicated in the pathogenesis of osteoporosis, particularly in individuals older than 60 years. Therefore, cortical bone is thought to be a second target in the prevention and treatment of osteoporotic fractures^40–44^. These findings imply that trabecular bone and cortical bone should be treated as two different compartments in pathological conditions, such as osteoporosis.

T1DM is a common pathological condition that reduces the bone mass and increases the risk of bone fracture. However, whether T1DM differentially affects trabecular bone and cortical bone remains unclear. A previous study reported that the trabecular volumetric bone mineral density (vBMD) was reduced to a greater extent than the cortical vBMD at both the radius and tibia in adult T1DM patients with diabetic microvascular disease^45^, whereas another study indicated no significant difference in the extent of reduced bone mineral content between the trabecular bone and cortical bone in male adolescents with T1DM^46^. In addition, a recent cross-sectional study showed that adult T1DM patients presented with a cortical bone size deficit, which might be attributed to long-term glycemic variability, as well as a higher bone marrow fat content^47^. The potential causes for these conflicting results may be the disease duration and the age of onset, as well as the impact of blood glucose fluctuations.

In the present study, we found that bone loss was more striking in the trabecular bone than that in the cortical bone in mice with STZ-induced T1DM and that the primary cause of this difference may be the greater impairment of bone formation in the trabecular bone than that in the cortical bone.

We subsequently investigated the underlying mechanism. The circulating levels of IGF1 and the expression of IGF-1R in bone tissues (mainly in osteoblasts) are significantly lower in STZ-induced T1DM rats or patients with T1DM than those in healthy controls^48,49^.

The reduced IGF-1R in osteoblasts inhibits AKT phosphorylation, which could inhibit GSK3β phosphorylation. Because GSK3β also acts as a key regulator of canonical WNT signaling, the inhibition of GSK3β phosphorylation causes the degradation of β-catenin in osteoblasts, which leads to impaired bone formation^11,18,50^. Moreover, a recent study indicated that the serum levels of the WNT signaling inhibitor Dickkopf-1 were higher in children and adolescents with T1DM than those in controls^51^, which may be related to a greater inhibitory signal of the WNT/β-catenin system on preosteoblast and osteoblast cells.

In this study, the expression of IGF-1R was lower in the trabecular bone than that in the cortical bone of T1DM mice within the same apparent volume of bone mass, leading to the different downregulated expression levels of β-catenin in the two bone compartments through AKT/GSK3β pathway inhibition. Moreover, the expression of Dickkopf-1 was increased to a greater extent in the trabecular bone than that in the cortical bone of T1DM mice. These differences ultimately result in a greater level of bone loss in trabecular bone than those in cortical bone.

Further experiments showed that the activation of β-catenin in T1DM mice also differentially affected the trabecular and cortical bone. Although a significantly increased bone mass and improved bone strength were identified in the trabecular bone of T1-CA mice, only a slight increase occurred in the bone mass of the cortical bone. Unexpectedly, a higher cortical porosity and increased bone resorption were identified in the cortical bone of the T1-CA mice than that in the T1DM mice. Because a small increase in porosity disproportionately reduces the resistance to bending^44,52,53^, cortical porosity is a major determinant of bone strength and fracture risk in the cortical bone. Therefore, an increased cortical porosity abrogates the positive effect of the increased cortical bone mass on the bone strength after β-catenin activation in T1DM mice, and the bone strength ultimately remains unchanged.

The potential mechanisms that underlie the increased bone resorption in the cortical bone of T1-CA mice were subsequently investigated. RANKL and OPG are recognized as key regulators of osteoclastogenesis^54^. However, there were no significant differences in the expression trends of RANKL or OPG or the RANKL/OPG ratio between the cortical and trabecular bone of the T1-CA mice. Therefore, the different effects on the osteoclasts in the two bone types were independent of the RANKL/OPG system.

Recent studies have shown that osteoblast-derived WNT16 is an important regulator of osteoclastogenesis, accomplished by its inhibitory effect on the proliferation and maturation of osteoclast progenitors^30,55^. In addition, endogenous WNT16 is highly expressed in cortical bone; thus, WNT16-deficient mice exhibited a low thickness and a high porosity only in cortical bone, whereas trabecular bone is not affected^30^. Here, we confirmed a higher WNT16 expression in the cortical bone than that in the trabecular bone of T1DM mice, and after β-catenin activation, the WNT16 expression significantly decreased in the cortical bone of the T1-CA mice, which could be an important cause of the increased number of osteoclasts. Moreover, although WNT16 expression also decreased in the trabecular bone of the T1-CA mice, the bone resorption was not increased and the anabolic effect of β-catenin activation in trabecular bone was not affected, which could be due to the relatively low expression of endogenous WNT16 in trabecular bone.

WNT5a regulates osteoclastogenesis, in part, through interaction with WNT16 and is also highly expressed in cortical bone^29,55^. However, there was no significant change in the WNT5a expression before and after β-catenin activation, which suggests that WNT5a does not contribute to the increase in osteoclast activity identified in the cortical bone of T1-CA mice. Therefore, we postulate that downregulated WNT16 expression is the major cause of the unexpected increase in cortical porosity and bone resorption after β-catenin activation in the context of T1DM.

This study had several limitations. First, increased oxidative stress and the accumulation of AGEs in T1DM have been reported to increase the apoptosis of osteoprogenitor cells and impair osteoblast activities^56–58^. Therefore, whether these two factors contribute to the differences identified in trabecular and cortical bone and their effects on WNT/β-catenin signaling in T1DM mice require further investigation. Second, other signaling pathways may also regulate the differential effects of T1DM on trabecular and cortical bone and may interact with WNT/β-catenin signaling. A recent study reported that the inactivation of FOXOs only ameliorated the loss of trabecular bone, and not the loss of cortical bone, in STZ-treated mice^59^. It is well established that FOXOs prevent the association between β-catenin and the TCF and LEF transcription factors and the subsequent WNT/β-catenin transcriptional activity^60^. Further studies are required to clarify the contributions of these factors and their signaling pathways to the differences between trabecular bone and cortical bone in T1DM.

In conclusion, T1DM may differentially affect trabecular and cortical bone, and in general, trabecular bone loss is greater than cortical bone loss in this disease. The present study has shown a novel intervention that activates WNT/β-catenin signaling, which may provide a therapeutic opportunity to improve bone strength in T1DM patients. However, it exerts different effects on cortical bone, causing an unexpected increase in the bone porosity, which impairs the cortical bone strength. Therefore, the different outcomes for trabecular bone and cortical bone should be appropriately addressed and warrant further research.

## Electronic supplementary material

supplemental Figure legends

Supplemental Table 1

supplemental Figure 1

supplemental Figure 2

supplemental Figure 3

supplemental Figure 4
