# Supplementary material for: Differential effects of type 1 diabetes mellitus and subsequent osteoblastic β-catenin activation on trabecular and cortical bone in a mouse model
Source: Exp Mol Med. 2018 Dec 5;50(12):158. doi: 10.1038/s12276-018-0186-y (PMC6281645; doi:10.1038/s12276-018-0186-y)
Supplement: Supplementary file 1 — supplemental Figure legends [file 12276_2018_186_MOESM1_ESM.docx]

**Supplemental Figure 1** General parameters including body weight and femur length. n=9. Data are expressed as the mean ± SD. **P* < 0.05 versus Control group by an unpaired t-test. NS, not significant, *P*>0.05.

**Supplemental Figure 2** β-galactosidase staining for the site-specificity of Col1-3.2kb-Cre^ERTM^ activity in trabecular and cortical bone. Scale bars, 40μm.

**Supplemental Figure 3** Representative images of β-catenin immunohistochemical staining in osteoblasts. Black arrow heads point to osteoblasts with β-catenin positive staining. Scale bars, 50μm.

**Supplemental Figure 4** Representative images of H&E stained longitudinal sections of the fifth lumbar vertebrae. Scale bars, 100μm.
