# Supplementary material for: Differential effects of type 1 diabetes mellitus and subsequent osteoblastic β-catenin activation on trabecular and cortical bone in a mouse model
Source: Exp Mol Med. 2018 Dec 5;50(12):158. doi: 10.1038/s12276-018-0186-y (PMC6281645; doi:10.1038/s12276-018-0186-y)
Supplement: Supplementary file 2 — Supplemental Table 1 [file 12276_2018_186_MOESM2_ESM.docx]

**Table 1. Sequences for real-time PCR primers**

| Gene | Forward (5’ to 3’) | Reverse (5’ to 3’) |
| --- | --- | --- |
| ALP | TGACCTTCTCTCCTCCATCC | CTTCCTGGGAGTCTCATCCT |
| OSX | GCAACTGGCTAGGTG-GTGGTC | GCAAAGTCAGATGGGTAAGTAGGC |
| β-catenin | GATTTCAAGGTGGACGAGGA | CACTGTGCTTGGCAAGTTGT |
| LEF-1 | TGGCATCCCTCATCCAGCTAT | TGAGGCTTCACGTGCATTAGG |
| TCF | CCTCTCTGGCTTCTACTCCCT | CAGCCTGGGTATAGCTGCATGT |
| Axin2 | AGTCAGCAGAGGGACAGGAA | CTTCGTACATGGGGAGCACT |
| IGF-1R | GTGCTGTACGCCTCTGTGAA | TTGCAGCCTCATTCACTGTC |
| RunX2 | GGAATGATGAGAACTA | ACCGTCCACTGTCACTTT |
| Osteocalcin | TGCTTGTGACGAGCTATCAG | GAGGACAGGGAGGATCAAGT |
| OPG | GTCCCTTGCCCTGACTACTCT | GACATCTTTTGCAAACCGTGT |
| RANKL | GCTGGGCCAAGATCTCTAAC | GTAGGTACGCTTCCCGATGT |
| Wnt16 | GAGCTGTGCAAGAGGAAACC | TCCTGTGGTGTTTCTGATGG |
| Wnt5a | CCGCGAGCGGGAGCGCAT | GCCACATCAGCCAGGTTGTACACC |
| β-actin | CAGAGCCTCGCCTTTGCCGATCC | GGCCTCGTCGCCCACATAGGA |
