# Supplementary figures and images for: Differential effects of type 1 diabetes mellitus and subsequent osteoblastic β-catenin activation on trabecular and cortical bone in a mouse model
Source: Exp Mol Med. 2018 Dec 5;50(12):158. doi: 10.1038/s12276-018-0186-y (PMC6281645; doi:10.1038/s12276-018-0186-y)

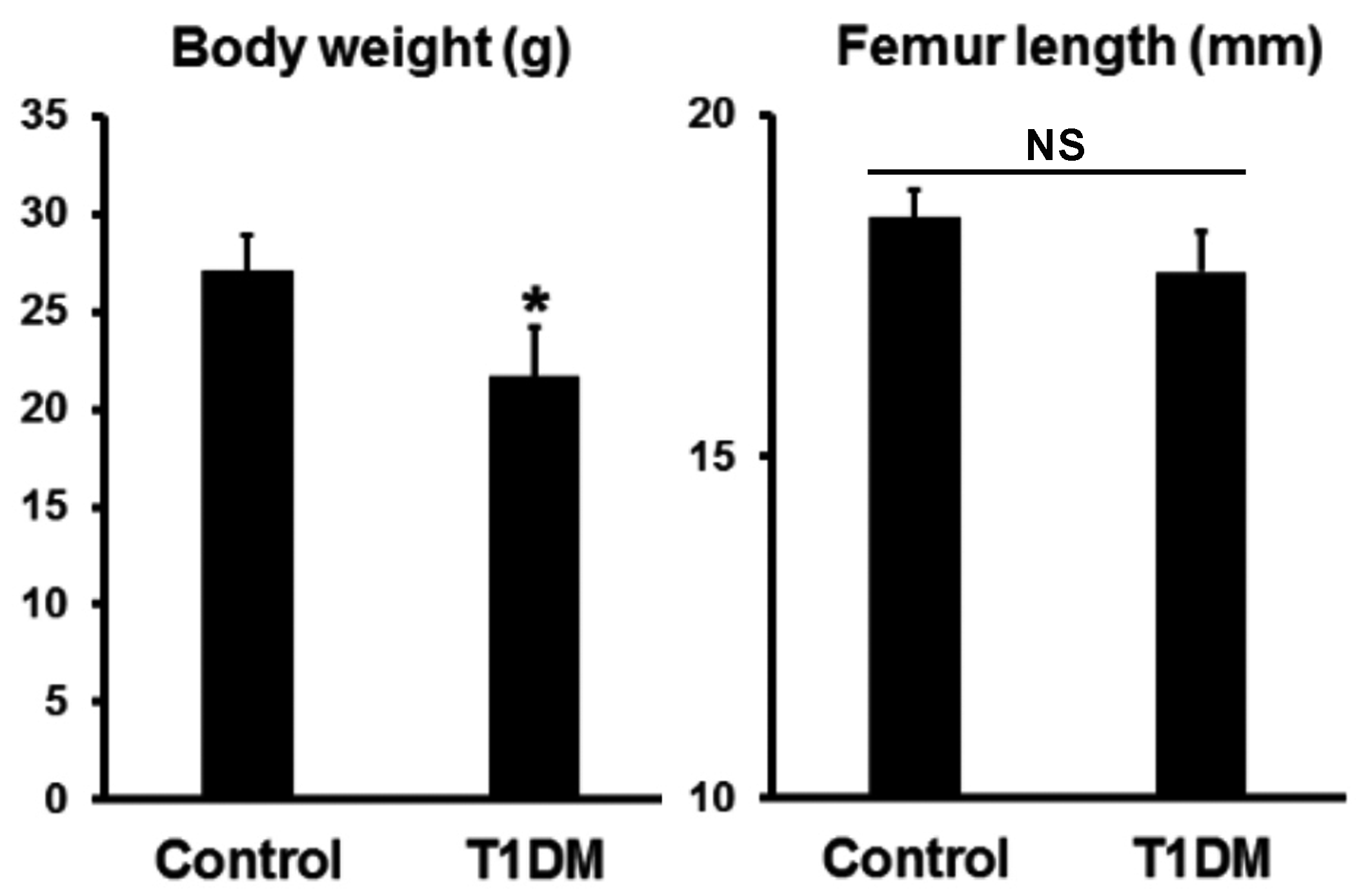

Supplement: Supplementary file 3 — supplemental Figure 1 [file 12276_2018_186_MOESM3_ESM.tif]

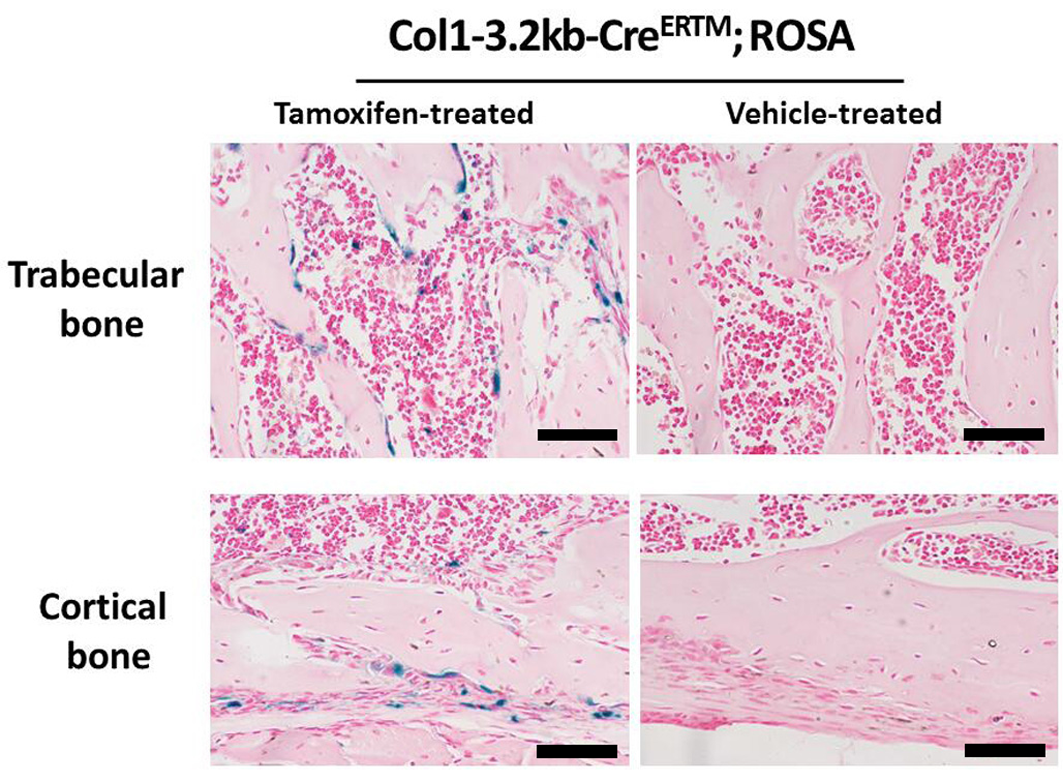

Supplement: Supplementary file 4 — supplemental Figure 2 [file 12276_2018_186_MOESM4_ESM.tif]

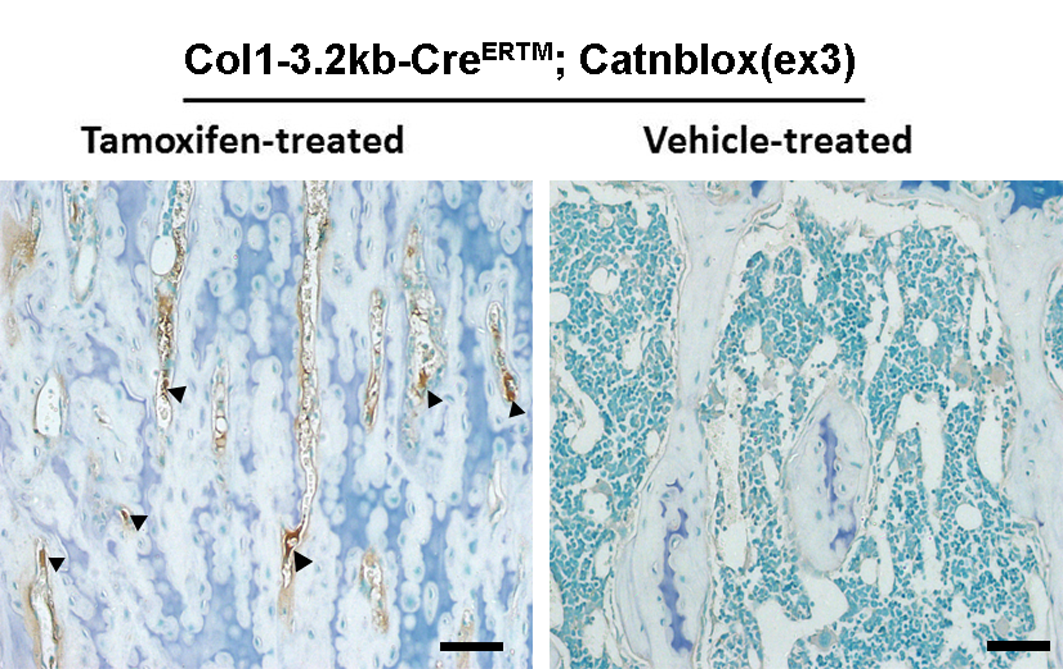

Supplement: Supplementary file 5 — supplemental Figure 3 [file 12276_2018_186_MOESM5_ESM.tif]

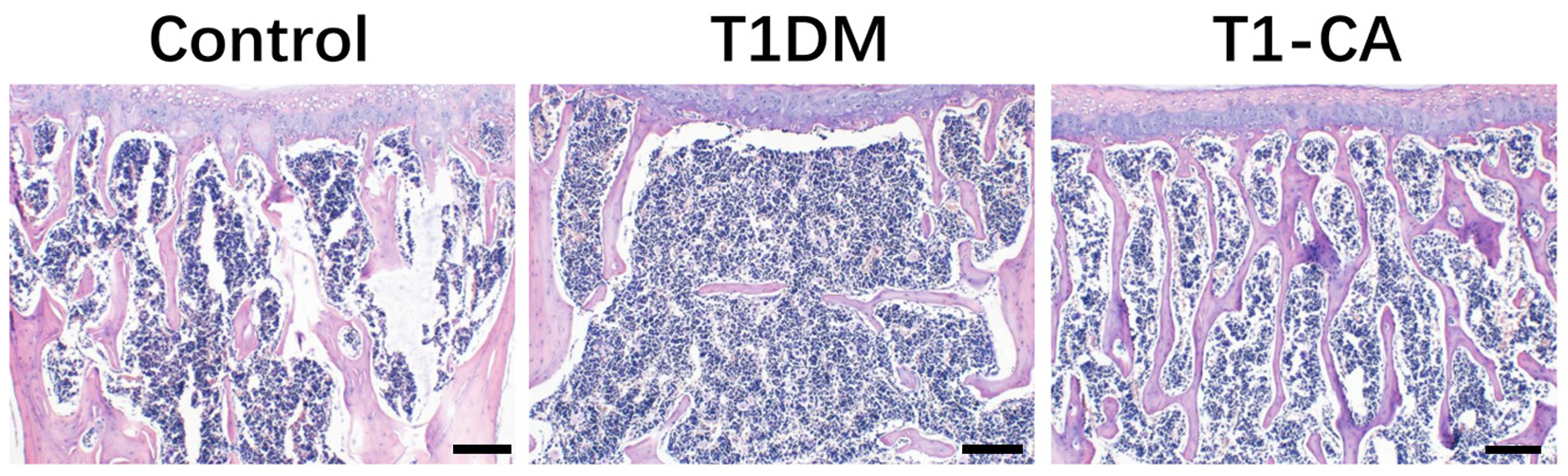

Supplement: Supplementary file 6 — supplemental Figure 4 [file 12276_2018_186_MOESM6_ESM.tif]
